# Pharmacological blockade of TRPA1 inhibits mechanical firing in nociceptors

**DOI:** 10.1186/1744-8069-5-19

**Published:** 2009-04-21

**Authors:** Patrick C Kerstein, Donato del Camino, Magdalene M Moran, Cheryl L Stucky

**Affiliations:** 1 Department of Cell Biology, Neurobiology and Anatomy, Medical College of Wisconsin, 8701 Watertown Plank Road, Milwaukee, WI 53226, USA; 2 Hydra Biosciences, Inc. 790 Memorial Drive, Cambridge, MA 02139, USA

## Abstract

**Background:**

TRPA1 has been implicated in both chemo- and mechanosensation. Recent work demonstrates that inhibiting TRPA1 function reduces mechanical hypersensitivity produced by inflammation. Furthermore, a broad range of chemical irritants require functional TRPA1 to exert their effects. In this study we use the *ex-vivo *skin-nerve preparation to directly determine the contribution of TRPA1 to mechanical- and chemical-evoked responses at the level of the primary afferent terminal.

**Results:**

Acute application of HC-030031, a selective TRPA1 antagonist, inhibited all formalin responses in rat C fibers but had no effect on TRPV1 function, assessed by capsaicin responsiveness. Genetic ablation experiments corroborated the pharmacological findings as C fibers from wild type mice responded to both formalin and capsaicin, but fibers from their TRPA1-deficient littermates responded only to capsaicin. HC-030031 markedly reduced the mechanically-evoked action potential firing in rat and wild type mouse C fibers, particularly at high-intensity forces, but had no effect on the mechanical responsiveness of Aδ fiber nociceptors. Furthermore, HC-030031 had no effect on mechanically-evoked firing in C fibers from TRPA1-deficient mice, indicating that HC-030031 inhibits mechanically-evoked firing via a TRPA1-dependent mechanism.

**Conclusion:**

Our data show that acute pharmacological blockade of TRPA1 at the cutaneous receptive field inhibits formalin-evoked activation and markedly reduces mechanically-evoked action potential firing in C fibers. Thus, functional TRPA1 at sensory afferent terminals in skin is required for their responsiveness to both noxious chemical and mechanical stimuli.

## Background

Transient receptor potential ankyrin 1 (TRPA1) is a member of the TRP superfamily of ion channels, which have been implicated in multiple somatosensory modalities including thermosensation [1-3], osmosensation [4] and mechanosensation [5,6]. Like other TRP channels, TRPA1 is predicted to have six transmembrane domains, a pore region that passes Na^+ ^and Ca^2+^, and is thought to form tetramers in native sensory neurons. Among sensory neurons, TRPA1 is expressed by a subset (approximately 50%) of small-diameter neurons that express the capsaicin receptor TRPV1 and are predominantly unmyelinated (C fiber) nociceptors [7-10]. Consistent with its expression in nociceptors, TRPA1 is activated by pungent or irritating chemicals, including mustard oil, cinnamon oil, raw garlic extracts, environmental irritants such as exhaust fumes, isocyanates and tear gas, and endogenous signaling molecules like 15delta-PGJ_2 _[11-17]. Exposure to each of these elicits a painful burning or prickling sensation in humans. TRPA1 has also been proposed as a direct cold transducer, however, the evidence in both heterologous cell lines and *in vivo *is still controversial [7,11,14,18-20].

A unique structural feature is that TRPA1 is the only mammalian TRP channel that contains an extended ankyrin repeat domain in the N-terminus. Ankyrin repeats have been hypothesized to tether ion channels to cytoskeletal elements and contribute to mechanical gating of the channel [21,22]. Evidence from behavioral studies across multiple species indicates that TRPA1 is involved in mechanotransduction. *Drosophila *larva deficient in *painless*, an invertebrate TRPA homologue, have decreased mechanical nociception [6]. *C. elegans *with mutations in TRPA1 fail to show head withdrawal or stop feeding after nose touch, and exhibit exaggerated head movements during foraging behavior [23]. Mice with a deletion in the pore domain of TRPA1 have decreased behavioral responses to intense mechanical force in the noxious range [18]. Recent data from our laboratory has shown that TRPA1 contributes to mechanotransduction at the level of primary afferent terminals. Recordings in skin nerve preparations from TRPA1 deficient mice and their wild type littermates revealed that in the absence of TRPA1, C fiber and Aδ fiber nociceptors from non-injured skin fire substantially fewer action potentials in response to sustained, intense force [24], indicating that TRPA1 is necessary for nociceptors to attain their normal firing rate to mechanical stimuli.

Despite the wealth of data examining the effects of genetic ablation of TRPA1, few studies have probed the effects of acutely inhibiting TRPA1. Pharmacological inhibition of TRPA1 points to a broader role for the channel in mechanosensation than is indicated by genetic studies. Whereas mice lacking TRPA1 show no deficit in mechanical hypersensitivity induced by inflammation, the TRPA1 antagonist AP-18 profoundly reduces this hypersensitivity. These effects of AP-18 are only observed in mice that express functional TRPA1 [25]. Given the differences observed between chronic and acute disruption of TRPA1, we sought to investigate the role of TRPA1 in cutaneous chemo- and mechanosensation by using a selective antagonist, HC-030031. HC-030031 inhibits formalin-induced pain, prostaglandin-induced activation of sensory neurons, cigarette smoke-induced inflammation, and CFA-induced mechanical hyperalgesia, by direct inhibition of the TRPA1 channel [15,26-28]. We utilized HC-030031 in combination with the saphenous skin-nerve preparation where the functional properties of cutaneous sensory neuron terminals *in situ *can be assessed. We show here that acute pharmacological inhibition of TRPA1 substantially decreases the responsiveness of nociceptors to both formalin and mechanical force. In contrast, C fiber responses to capsaicin, as well as A-mechanoreceptor responses to force, were unperturbed. HC-030031 also failed to affect responses in mice that lack functional TRPA1. Taken together, we conclude that TRPA1 plays a significant role in the normal detection of noxious stimuli and that pharmacological inhibition of TRPA1 could have significant utility as a treatment for mechanical pain.

## Methods

### Animals

Male Sprague-Dawley rats (Charles River Laboratories, Wilmington, MA), ages 8 – 16 weeks old (200 – 400 g body weight), were used for skin-nerve recordings of mechanically- or chemically-evoked action potentials in cutaneous afferent fibers. In some experiments male and female mice lacking the *Trpa1 *gene and their wild type littermates [18], ages 11–30 weeks old, were used for mechanically-evoked action potential recordings.

### Chemicals

The TRPA1 channel antagonist, HC-030031 (Hydra Biosciences, Cambridge, MA) was dissolved in dimethyl sulfoxide (DMSO; stock 99.9%) and diluted to a final working concentration of 100 μM with synthetic interstitial fluid (SIF) containing (in mM): 123 NaCl, 3.5 KCl, 0.7 MgSO_4_, 1.7 NaH_2_PO_4_, 2.0 CaCl_2_, 9.5 sodium gluconate, 5.5 glucose, 7.5 sucrose, and 10 HEPES, 290 ± 3 mOsm, at pH 7.45 ± 0.05. For a vehicle control, DMSO was diluted in SIF buffer to the same final concentration of DMSO (1%) in the solution with antagonist. A 10% formalin stock solution containing 4% wt/vol formaldehyde, 0.4% wt/vol sodium phosphate monohydrate, 0.65% sodium phosphate anhydrous, and 1.5% wt/vol methanol (Fisher Scientific, Fair Lawn, NJ) was diluted with SIF buffer to a 0.010% formalin working solution. For experiments with capsaicin, a 10 mM stock solution was made in 1-methyl-2-pyrrolidinone, and diluted with SIF buffer to a working concentration of 10 μM. These working concentrations were based on previous skin nerve recordings in rodents because they elicit near maximal action potential responses from C fibers without inducing significant desensitization or non-specific effects [26,29,30]. The SIF buffer and all chemical-containing solutions were made fresh daily.

### Skin-nerve preparation

The saphenous skin-nerve preparation was used to record from the terminals of primary afferent fibers *in situ*. All experiments and data analyses were performed with the operator blind to genotype or drug treatment. Mice and rats were briefly anesthetized with isoflurane, and sacrificed by either cervical dislocation (mice) or pneumothorax (rats). The saphenous nerve and skin from the medial dorsum of the hind paw were dissected free and placed corium side up into a bath superfused with oxygen-saturated SIF buffer at physiological skin temperature, 32.0 ± 0.5°C. The nerve was desheathed and teased into thin filaments, from which extracellular recordings of action potentials were made. A mechanical search stimulus (finely-tipped glass rod) was applied systematically across the skin preparation to locate single afferent fibers. Following action potential identification (signal: noise ratio >3), the surrounding tissue was probed to locate the most sensitive spot of the receptive field. Calibrated von Frey filaments (US Neurologicals, Kirkland, WA, USA) were used to find the mechanical force threshold. An electrical stimulus was used to determine the conduction velocity by inserting a Teflon-coated steel needle electrode (2 MΩ impedance, un-insulated tip diameter of 10 μm) into the most mechanically-sensitive spot of the receptive field and square-wave pulses (500 μs, 6 mA) were used to stimulate the fiber electrically.

Fiber types were categorized by conduction velocity. In agreement with previous studies from mouse and rat [24,30,31], units conducting slower than 1.2 m/s were classified as unmyelinated C fibers; those conducting between 1.2 and 10 m/s were classified as thinly myelinated Aδ fibers. This study utilized only Aδ fibers that exhibited slowly adapting responses to sustained force and were thus classified as A-mechanoreceptors (AM).

### Chemical treatment and stimulation of isolated receptive fields

In order to treat the receptive field of the nociceptor of interest with the HC-030031 antagonist or vehicle, and to stimulate the terminals with formalin or capsaicin, a glass ring sealed to the skin was used to isolate the receptive field from fluid in the rest of the chamber. The SIF buffer was removed from inside the ring, a seal was confirmed and either HC-030031 or vehicle was added to the ring for 10 minutes. During incubation, oxygen was gently bubbled into the ring to maintain the oxygen level and to continuously mix the solution inside the ring.

For fibers tested with mechanical stimuli, after the 10 min incubation, each fiber was tested with quantitative force in the presence of the antagonist or vehicle. Sustained force was applied via a custom-made feedback-controlled computer-driven stimulator which controlled a cylindrical probe (tip diameter 0.60 mm) placed perpendicular to the most mechanically-sensitive spot of the receptive field. Each fiber was tested with square-wave mechanical force in ascending order of 5, 10, 20, 40, 100, and 150 mN (10 sec each; 2 min interstimulus interval).

Another group of fibers was tested with formalin and capsaicin. After the 10 min incubation with HC-030031 or vehicle, the solution in the ring was removed and replaced with formalin (0.010%) and action potentials were quantified for 2 min beginning with the onset of the chemical addition. Formalin was then replaced with HC-030031 or vehicle for a 4 min washout preceding the 2 min capsaicin (10 μM) treatment and action potential quantification. Formalin and capsaicin solutions contained HC-030031 or vehicle at the concentrations indicated.

### Fiber Analysis

Mechanically-, chemically- or electrically-evoked action potential wave forms were saved on an oscilloscope for comparison of shape and profile to ensure that a single unit of interest was stimulated via all modalities. All data were collected using a Powerlab 4.0 system and Chart 5 software (ADInstruments, Colorado Springs, CO, USA) and saved for offline analysis. Action potentials were discriminated and counted offline using a spike histogram software extension. In rare instances where fibers exhibited ongoing firing, a baseline firing rate was measured during the 10 sec preceding each mechanical stimulus application. Mechanically-evoked action potential counts were determined by subtracting the baseline firing rate from the total action potentials that occurred during each 10 sec mechanical stimulus. Similarly, in experiments with chemical stimuli, for fibers that exhibited ongoing firing, a baseline firing rate was quantified during the 2 min preceding the application of the chemical. The baseline firing rate was subtracted from the total action potentials that occurred during the chemical stimulus.

### Statistical Analysis

Values for conduction velocity, electrical threshold, and mechanically- or chemically-evoked action potentials are given as the mean ± SEM. Von Frey thresholds are given as the median, and 25^th ^and 75^th ^percentiles. A 2-way repeated measures analysis of variance (ANOVA) was used to compare mechanically-evoked action potentials generated over the entire force continuum between HC-030031- and vehicle-treated groups. For comparison of action potentials per sec over a 10 sec sustained force, a 2-way ANOVA was used. Percentages of total responding fibers were analyzed via a Fisher's exact test. Comparisons of the chemically-evoked action potentials between HC-030031- or vehicle-treated fibers were made using an unpaired Student's t-test. To determine the effect of HC-030031 or vehicle on the conduction velocity, electrical threshold, or von Frey threshold of fibers, the data collected before and after the 10 min chemical treatments were compared using a paired Student's t-test for parametric data (conduction velocity and electrical threshold) or Kruskal-Wallis test for non-parametric data (von Frey thresholds).

## Results

### HC-030031 blocks formalin, but not capsaicin, stimulation in rat C fibers

**Figure 1:**
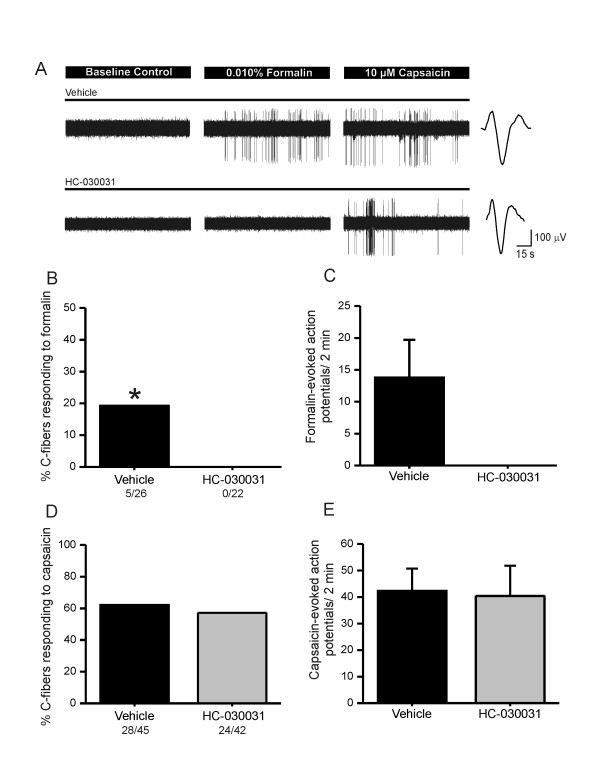
**HC-030031 inhibits formalin response but not capsaicin response in rat C fibers**. **A**) An example of a C fiber from rat responding to formalin (0.010%) and then capsaicin (10 μM) after a 10 min incubation of the receptive field with either vehicle (1% DMSO; top; conduction velocity: 0.394 m/s; von Frey threshold: 14.6 mN) or HC-030031 (100 μM; bottom; conduction velocity: 0.298 m/s; von Frey threshold: 20.1 mN). Action potential waveform is shown at the right of each trace. A 4 min washout was given between the formalin and capsaicin tests, and the receptive field was treated with either vehicle or HC-030031 during the washout period. **B**) Percentage of total C fibers responding to formalin after a 10 min treatment with vehicle or HC-030031 (100 μM; p = 0.05; Fisher's exact test). **C**) The average formalin-evoked action potentials over 2 min after 10 min treatment with vehicle or HC-030031. **D**) Percentage of total C fibers responding to capsaicin after a 10 min treatment with vehicle or HC-030031 (100 μM; p > 0.05; Fisher's exact test). **E**) The mean capsaicin-evoked action potentials over 2 min after 10 min treatment with vehicle or HC-030031 (p > 0.05; Student's t-test).

Previous reports indicate that rats treated with HC-030031 show a significant deficit in formalin evoked pain behaviors [26]. To address whether this deficit occurs at the level of the nociceptor as was predicted based on calcium imaging experiments, we examined C fiber responsiveness in the presence and absence of HC-030031. In the presence of the vehicle, 19% of C fibers showed responses to a low concentration (0.010%) of formalin (Figure 1A, top; 1B) and exhibited an average of 13.8 ± 5.9 action potentials over 2 min (Figure 1C). Application of HC-030031 blocked all formalin evoked action potentials in rat C fibers (Figure 1A–C).

All C fibers that responded to formalin (5/5) were also sensitive to the TRPV1 agonist, capsaicin. This is consistent with other studies showing that C fiber neurons that express TRPA1 also express the capsaicin receptor TRPV1 [7,11]. To determine whether the inhibitory effect of HC-030031 was specific for inhibition of TRPA1 but not other ion channels in these neurons, we determined whether HC-030031 affected the responsiveness of C fibers to the TRPV1 agonist, capsaicin. After a 10 min treatment with vehicle, 62% of rat C fibers responded to a high concentration (10 μM) of capsaicin (Figure 1D) with a typical barrage of spikes (Figure 1A, top) averaging 42.3 ± 8.4 action potentials over 2 min (Figure 1E). After treatment with HC-030031 (100 μM) for 10 min, capsaicin (10 μM) evoked a similar barrage of action potentials (Figure 1A, bottom). HC-030031 had no effect on the percentage of C fibers that responded to capsaicin (57%; Figure 1D) or the number of evoked action potentials (40.4 ± 11.4 action potentials/2 min; Figure 1E). This proportion of C fibers that is sensitive to capsaicin is highly consistent with other studies in both rats and mice [29,31]. There was no difference in the average conduction velocity or median von Frey threshold of HC-030031- and vehicle treated groups (not shown). These data indicate that this regimen of treatment with HC-030031 does not inhibit TRPV1 channels in cutaneous C fiber terminals *in situ *and provide support that HC-030031 selectively inhibits TRPA1 channels in the peripheral terminals of these unmyelinated nociceptors in this paradigm.

### C fibers have normal activation via capsaicin, but not formalin, in TRPA1-deficient mice

**Figure 2:**
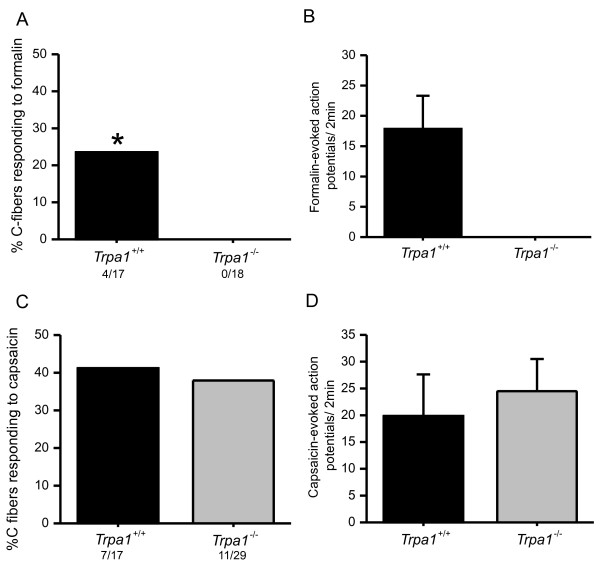
**Formalin and capsaicin responses of C fibers in TRPA1 wild type and knockout mice**. **A**) Percentage of total C fibers responding to formalin in *Trpa1 *+/+ or *-/- *mice (p = 0.046; Fisher's exact test). **B**) The average formalin-evoked action potentials over 2 min in C fibers from *Trpa1 *+/+ or *-/- *mice. **C**) Percentage of total C fibers responding to capsaicin in *Trpa1 *+/+ or *-/- *mice (p > 0.05; Fisher's exact test). **D**) The average capsaicin-evoked action potentials over 2 min in C fibers from *Trpa1 *+/+ or *-/- *mice.

We next used TRPA1-deficient mice to test whether genetic disruption of TRPA1 leads to a loss in formalin response, similar to experiments with acute inhibition by HC-030031. C fibers from *Trpa1*^-/- ^mice completely lacked the capability of responding to formalin applied to their receptive terminals when compared to 24% of C fibers that responded in their wild type littermates (Figure 2A). Formalin evoked an average of 18.0 ± 5.3 action potentials over 2 min in C fibers from wild type mice (Figure 2B). These data parallel the acute inhibition of TRPA1 by showing that either genetic ablation or pharmacological inhibition of TRPA1 inhibits C fiber responsiveness to formalin.

We also tested the capacity of C fibers from *Trpa1*^-/- ^mice to respond to capsaicin. Similar to our finding in rats, we found that all formalin-sensitive C fibers in wild type mice were also capsaicin-sensitive (4/4 fibers). In addition, we found similar numbers of capsaicin sensitive fibers in wild type mice (41%) and *Trpa1*^-/- ^mice (38%; Figure 2C). Furthermore, capsaicin evokes a similar level of action potential firing in C fibers from wild type and *Trpa1*^-/- ^mice (Figure 2D). These data indicate that developmental disruption of TRPA1 does not affect capsaicin responsiveness.

### Pharmacologic blockade of TRPA1 decreases mechanically-evoked firing in rat C fiber nociceptors

**Figure 3:**
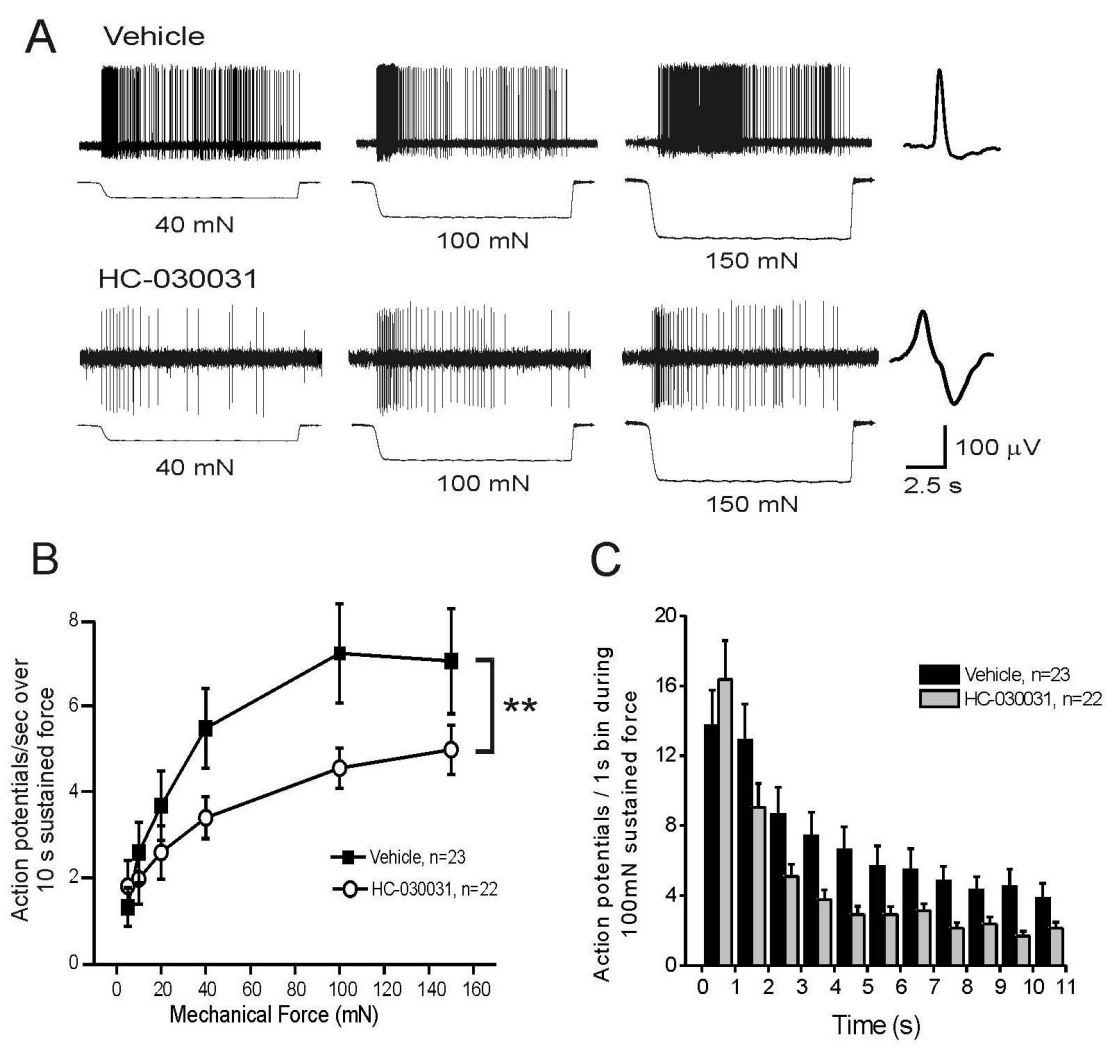
**HC-030031 inhibits mechanically-evoked firing in C fiber nociceptors**. **A**) Example of C fibers from rat responding to increasing sustained force (40 mN, 100 mN, 150 mN; 10 sec each) after a 10 min incubation of the receptive field with either vehicle (1% DMSO; top; conduction velocity: 0.632 m/s; von Frey threshold: 11.7 mN) or HC-030031 (100 μM; bottom; conduction velocity: 0.394; von Frey threshold: 20.1 mN). Action potential waveform is shown at the right of each trace. **B**) Average number of action potentials evoked per second in C fibers by a 10 sec, sustained mechanical force applied to the receptive field in increasing intensities (5 mN, 10 mN, 20 mN, 40 mN, 100 mN, 150 mN) in the presence of vehicle (solid squares) or HC-030031 (100 μM; open circles). Y-axis bars represent the SEM for each mean data point. HC-030031 significantly reduced the mechanically-evoked firing across all force intensities (p = 0.0029; 2-way repeated measures ANOVA). **C**) Adaptation rate of action potential firing during a 10 sec, 100 mN mechanical force in the presence of vehicle (black bars) or HC-030031 (100 μM; grey bars). HC-030031 significantly inhibited the firing rate after the first second (p = 0.0052; 2-way ANOVA).

**Figure 4:**
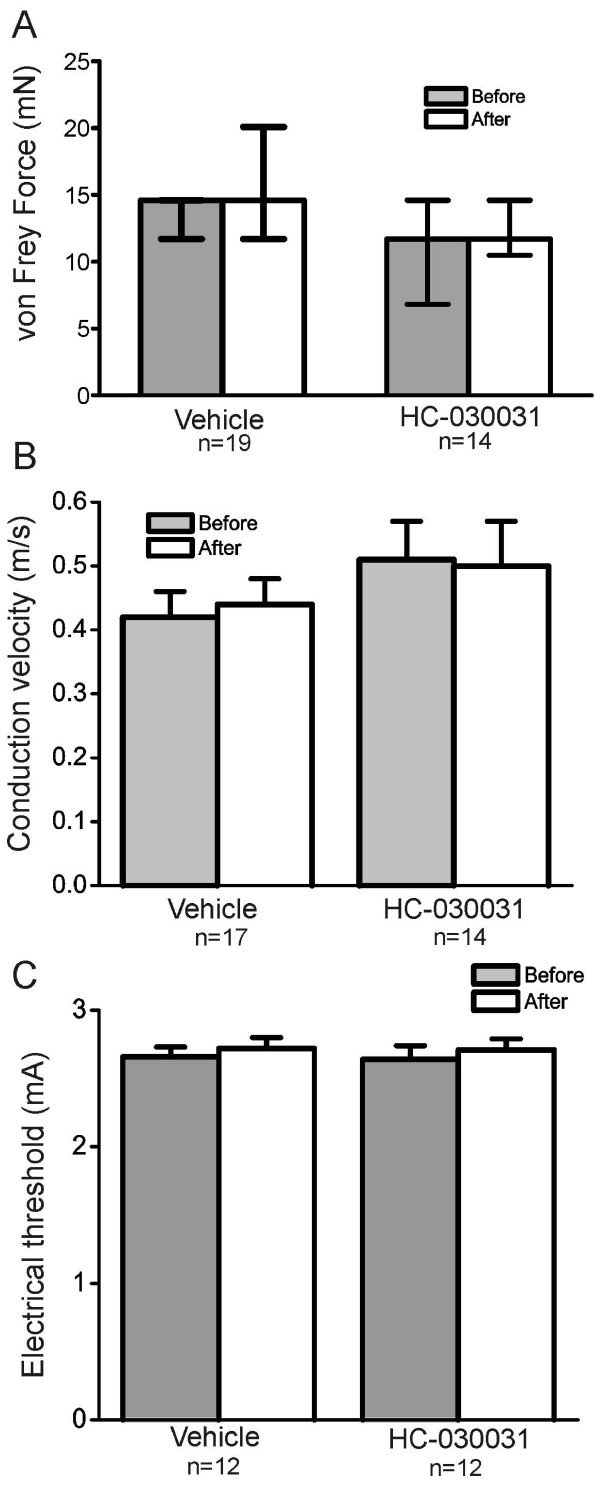
**HC-030031 has no effect on mechanical thresholds, conduction velocity, or electrical thresholds in C fibers**. **A**) Median von Frey thresholds in C fibers before and after 10 min treatment of the receptive field with vehicle or HC-030031 (100 μM). Y-axis bars represent the median and 25^th ^and 75^th ^percentiles for each group. **B**) Average conduction velocity of C fibers before and after 10 min treatment of the receptive field with vehicle or HC-030031. Y-axis bars represent the mean and SEM for each group. **C**) Average electrical threshold of C fibers before and after 10 min treatment of the receptive field with vehicle or HC-030031. Y-axis bars represent the mean and SEM for each group. The "after" measures were made in the presence of either vehicle or HC-030031.

TRPA1 inhibitors have also been shown to reduce mechanical hypersensitivity after inflammatory stimuli [25,28]. To address whether pharmacological blockade of TRPA1 at the level of the nociceptor terminal can mediate this effect, we studied mechanical responses in the saphenous skin-nerve preparation. We focused on C and Aδ fiber neurons since TRPA1 is highly expressed by a subset of small- and medium-diameter neurons, the majority of which have unmyelinated axons *in vivo*. We found that C fibers exhibit robust action potential firing in response to sustained force in the presence of vehicle (Figure 3A, top). In contrast, after 10 min treatment of the receptive field with the TRPA1 antagonist HC-030031 (100 μM), C fibers fired substantially fewer action potentials to sustained force (Figure 3A, bottom). For all C fibers tested, HC-030031 significantly reduced the average firing frequency compared to vehicle controls across a range of force intensities (Figure 3B; p = 0.0029; 2-way repeated measures ANOVA). The vehicle (1% DMSO) had no effect on mechanical firing rate in that the maximum firing rate in the presence of vehicle (100 mN: 7.27 ± 1.16 spikes/sec; n = 23) was not different from that in C fibers from naïve, untreated controls (100 mN: 6.98 ± 0.74 spikes/sec; n = 20). A more detailed analysis of the mechanical firing rate during a 10 sec sustained intense force (100 mN) indicates that in the presence of HC-030031, C fibers initially fired a normal number of action potentials, but showed significantly reduced firing after the first second and for the duration of the force (Figure 3C; p = 0.0052, 2-way ANOVA). This pattern of decreased firing after the first second in the presence of HC-030031 is evident in the example shown in Figure 3A. Treatment of the receptive fields with HC-030031 or vehicle for 10 min did not alter the median von Frey mechanical thresholds of C fibers (Figure 4A), nor did it alter their average conduction velocity (Figure 4B) or electrical thresholds (Figure 4C), when measures were re-tested in the presence of either chemical.

**Figure 5:**
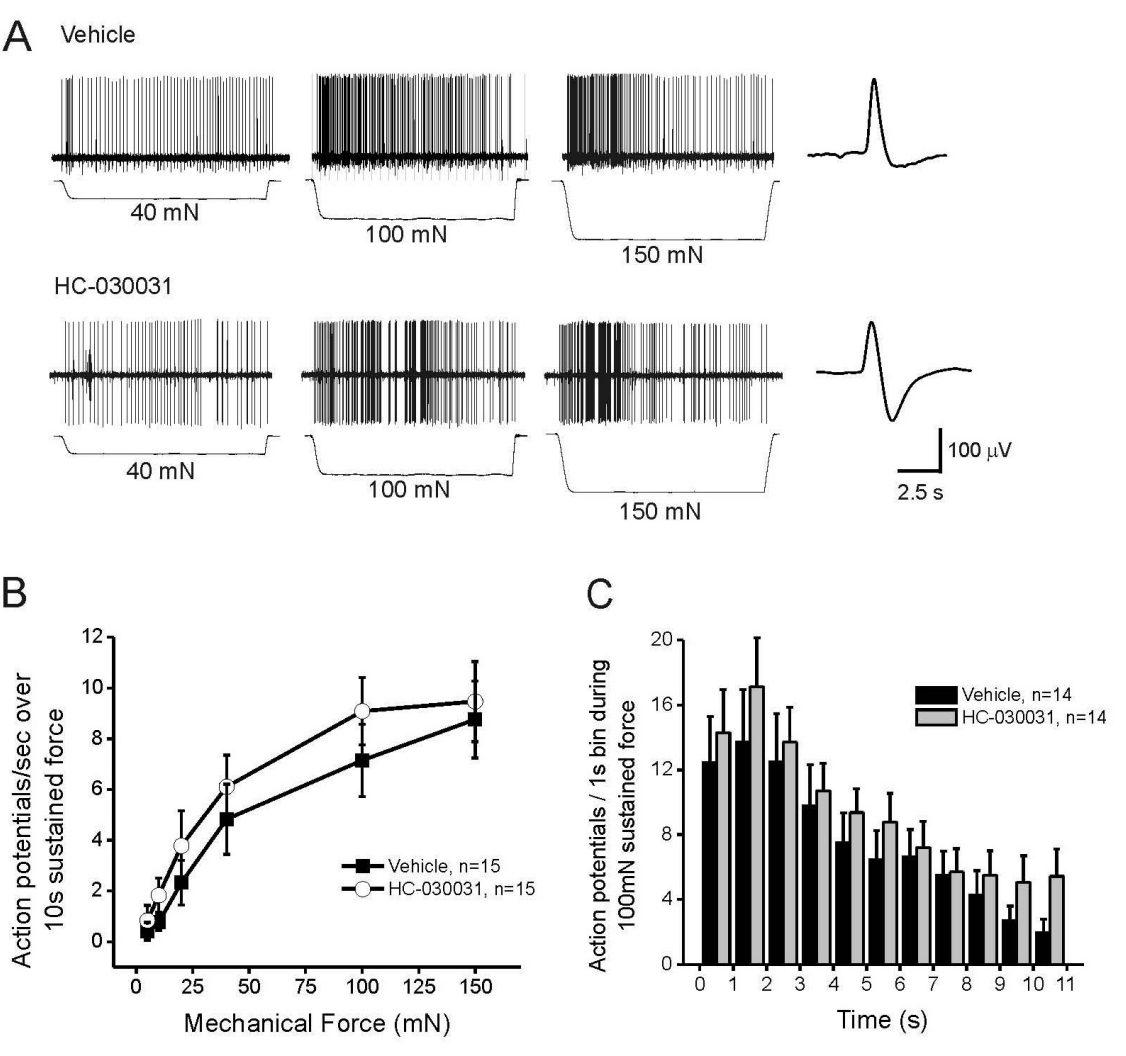
**HC-030031 has no effect on mechanical firing in A fiber nociceptors**. **A**) Example of an A-mechanoreceptor from rat responding to increasing sustained force (40 mN, 100 mN, 150 mN; 10 sec each) after a 10 min incubation of the receptive field with either vehicle (1% DMSO; top; conduction velocity: 8.65 m/s; von Frey threshold: 11.7 mN) or HC-030031 (100 μM; bottom; conduction velocity: 6.27 m/s; von Frey threshold: 14.6 mN). Action potential waveform is shown at the right of each trace. **B**) Average number of action potentials evoked per second in A-mechanoreceptors by a 10 sec, sustained mechanical force applied in increasing intensities in the presence of vehicle (solid squares) or HC-030031 (100 μM open circles). Y-axis bars represent the SEM for each mean data point. HC-030031 had no effect on the mechanical firing rate of A-mechanoreceptors at any stimulus intensity (p > 0.05; 2-way repeated measures ANOVA). **C**) Adaptation rate of action potential firing in A-mechanoreceptors during a 10 sec, 100 mN mechanical force in the presence of vehicle (black bars) or HC-030031 (100 μM; grey bars). HC-030031 had no effect on the adaptation rate (p > 0.05, 2-way ANOVA).

To determine whether HC-030031 selectively inhibits C fiber sensory neurons, we also examined the effect of HC-030031 on the firing rate of myelinated nociceptors (A-mechanoreceptors; AM fibers) since some small-medium diameter neurons that express TRPA1 could be A fiber nociceptors. Unlike C fibers, typical AM fiber responses showed robust firing to mechanical force in the presence of both the vehicle (Figure 5A, top) and HC-030031 (Figure 5A, bottom). Exposure to HC-030031 had no effect on mechanically-evoked action potential firing in AM fiber nociceptors at any force intensity (Figure 5B) and did not affect the firing rate during any part of a sustained 10 sec intense force (100 mN; Figure 5C).

### TRPA1 antagonist alters mechanically-evoked firing in mouse C fibers, but not in TRPA1-deficient littermates

**Figure 6:**
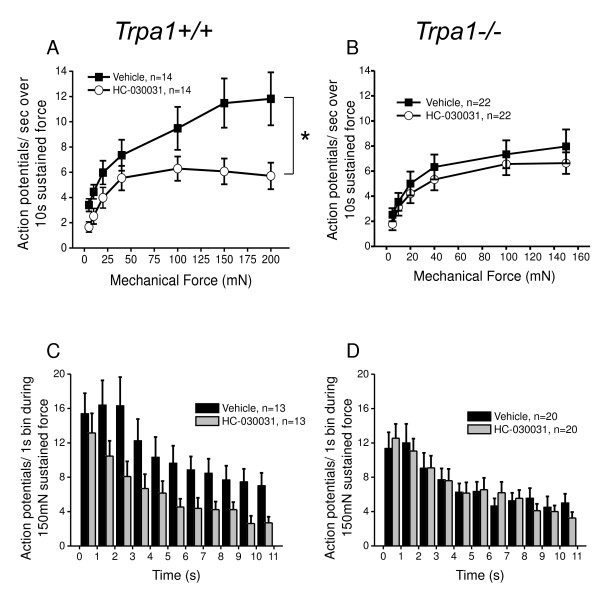
**HC-030031 inhibits mechanically-evoked action potentials in wild type mice but not in TRPA1-deficient mice**. **A) **C fibers from *Trpa1*^+/+ ^mice were tested with sustained (10 sec) mechanical force in increasing intensities from 5 to 200 mN in the presence of vehicle (solid squares) or HC-030031 (100 μM; open circles; p = 0.020, 2-way repeated measures ANOVA). **B) **C fibers from *Trpa1*^-/- ^mice were tested with sustained (10 sec) mechanical force in increasing intensities from 5 to 150 mN in the presence of vehicle (solid squares) or HC-030031 (100 μM; open circles; p > 0.05, 2-way repeated measures ANOVA). **C) **Adaptation rate of action potential firing in C fibers from *Trpa1*^+/+ ^mice during a 10 sec, 150 mN mechanical force in the presence of vehicle (black bars) or HC-030031 (100 μM; grey bars). HC-030031 significantly inhibited the firing rate after the first second (p = 0.024, 2-way ANOVA). **D) **Adaptation rate of action potential firing in C fibers from *Trpa1*^-/- ^mice during a 10 sec, 150 mN mechanical force in the presence of vehicle (black bars) or HC-030031 (100 μM; grey bars). HC-030031 had no effect on the adaptation rate in C fibers from *Trpa1*^-/- ^mice (p > 0.05, 2-way ANOVA).

To further determine whether the capacity of HC-030031 to inhibit mechanical firing in rat C fiber nociceptors was due specifically to inhibition of TRPA1 or to other off-target mechanisms, we investigated the effect of HC-030031 on mechanical firing in C fibers from TRPA1 deficient (*Trpa1*^-/-^) mice [18]. A 10 min treatment of C fiber receptive fields with HC-030031 in *Trpa1*^-/- ^mice had no effect on the mechanical firing rate compared to vehicle treated controls from *Trpa1*^-/- ^mice (Figure 6B). In addition, the firing rate throughout a 10 sec sustained force (100 mN) application did not differ between vehicle and HC-030031 treated C fibers (Figure 6D). To ensure that HC-030031 had the same inhibitory effect on the mechanical responses of C fibers in wild type mice as in rats, we tested C-fibers in wild type littermates from the TRPA1 strain. Similar to rat, HC-030031 significantly decreased the response to mechanical force in C fibers from *Trpa1*^+/+ ^mice compared to vehicle treated controls (Figure 6A, 2-way repeated measures ANOVA: p = 0.020). Furthermore, the firing rate throughout the 150 mN stimulus was significantly different after the first second between HC-030031- and vehicle-treated C fibers (Figure 6C, 2-way ANOVA: p = 0.024). These data indicate that HC-030031 reduces mechanical firing in cutaneous C fibers from normal wild type rodents by selectively inhibiting TRPA1 receptors at the level of the sensory terminals *in situ*.

## Discussion

TRPA1 is a cation channel that is robustly expressed on nociceptors. It responds to a broad range of chemical stimuli including an ever-expanding group of reactive molecules. Though it had been previously assumed that numerous effects of these reactive molecules were the result of non-specific tissue damage, emerging evidence suggests that many of these chemicals exert their acute effects in a TRPA1-dependent fashion. In addition to responding to chemical irritants, it has also been proposed that TRPA1 is involved in mechanosensation. While the role of TRPA1 in invertebrates has been fairly well established, it has been unclear if elimination of functional TRPA1 in mice reduces their sensitivity to mechanical stimuli.

Using a pharmacological approach, we examined the effects of acute, local inhibition of TRPA1 on transduction in cutaneous afferent terminals. Consistent with previous *in vivo *results [26], we show that functional TRPA1 in C fibers is required for responsiveness to low concentrations of formalin: both genetic ablation and pharmacologic inhibition abrogates these responses. In contrast, capsaicin responses in *Trpa1*^-/- ^mice are comparable to those observed in wild type mice. Similarly, responses to capsaicin in rat C fibers remain unaffected by treatment with the TRPA1 antagonist HC-030031. The specificity of the HC-030031-mediated inhibition of the formalin evoked responses underscores the likelihood that this antagonist exerts its effects by blocking TRPA1 rather than by affecting other aspects of neuronal firing.

We also examined the effects of acute inhibition of TRPA1 on mechanotransduction in the peripheral terminal. Inhibiting TRPA1 activity decreased the responsiveness of C fibers to high intensity mechanical force in both rat and mouse. Interestingly, the initial responses of the C fibers to low-intensity (5–20 mN) mechanical stimuli were relatively unperturbed in both species – instead, the firing to intense, sustained stimuli in the noxious range (>40 mN) was diminished. Responses of A-mechanoreceptors to both low-intensity and high-intensity sustained force were unaffected. Nociceptors were not affected by HC-030031 in mice that lack functional TRPA1, indicating that the effects of HC-030031 are mediated by on-target inhibition of TRPA1.

The TRPA1 antagonist failed to affect either the mechanical threshold or the initial (first second) response of C fibers to sustained mechanical force. Combined with the lack of clear evidence of direct mechanical activation of the recombinant mammalian TRPA1, these data raise the possibility that TRPA1 is not intrinsically mechanically sensitive, but instead serves as an amplifier or a signal integrator that is downstream of the bona fide mechanoreceptor(s). Given the broad range of molecules that can affect TRPA1 activity, a variety of possibilities, including local increases in intracellular calcium concentration [19,32,33], could explain how TRPA1 responds to activity of a mechanoreceptor. Alternatively, TRPA1 could be a part of a larger protein complex. For example, membrane integrins may contribute to detection of a mechanical stimulus and signal TRPA1 through a tyrosine kinase pathway. A similar complex has been recently proposed for the role of TRPV4 in mechanotransduction [34]. Nonetheless, our results clearly indicate that TRPA1 is required for the normal responsiveness of cutaneous C fiber terminals to intense force. Furthermore, two recent studies demonstrate that pharmacological inhibition of TRPA1 inhibits behavioral mechanical hyperalgesia after peripheral inflammation or neuropathic pain [25,28]. These data suggest that TRPA1 may play a more pronounced role in the sensitization of primary afferent neurons to mechanical stimuli after tissue injury, and TRPA1 may thereby provide a target for reducing mechanical pain following injury without abolishing mechanical sensitivity entirely.

Our results for Aδ nociceptors treated with the TRPA1 antagonist differ from a recent study in *Trpa1*^-/- ^mice where we used the same methods to assess mechanical sensitivity of Aδ fibers [24]. Whereas in the current study, Aδ nociceptors treated with the TRPA1 antagonist showed no change in mechanical firing, Aδ nociceptors from *Trpa1*^-/- ^mice in the prior study exhibited significantly reduced mechanical firing at intense forces above 100 mN [24]. Although it is possible that this discrepancy is due to developmental effects either at the molecular or cellular level, it is also possible that this difference results from a local versus a global reduction in TRPA1 function. For example, it is possible that normally, TRPA1 activation near the epidermal surface of the skin leads to the release of signaling molecules from non-neuronal skin cells that potentiate the A-mechanoreceptors. Such communication mechanisms have been proposed for heat activation of TRP channels in keratinocytes [3,35,36]. Indeed, a recent study from our laboratory shows that TRPA1 channels are widely expressed by keratinocytes that lie in close apposition to the free nerve terminals of both Aδ and C fibers in the epidermis [24]. Since the TRPA1 antagonist is applied locally to the inner dermal side of the skin in the current study, signaling factors may still be released from the epidermal keratinocytes that modulate mechanosensory terminals. Additional work will need to be performed to understand whether in A-mechanoreceptor or C fiber terminals, TRPA1 is acting in a cell autonomous fashion or via associated skin cells.

## Conclusion

In conclusion, we have shown that functional TRPA1 at the level of unmyelinated sensory afferent terminals in skin is required for their responsiveness to both noxious chemical and mechanical stimuli. Whereas the mechanisms by which sensory neurons respond to mechanical force are poorly understood, this study demonstrates that TRPA1 plays an important role in the ability of unmyelinated cutaneous nociceptors to respond to intense mechanical stimuli. Furthermore, our study highlights the importance of combining acute pharmacological inhibition with genetic studies. Thus, TRPA1 plays a significant role in the peripheral detection of noxious stimuli and pharmacological inhibition of TRPA1 may have significant utility as a treatment for acute or chronic pain that includes mechanical hypersensitivity.

## Competing interests

PCK and CLS declare no competing interests. DDC and MMM are employees and shareholders of Hydra Biosciences.

## Authors' contributions

PCK participated in the design of the study, carried out all of the experiments and analyses and helped draft the manuscript. CLS guided the design of the study and helped with interpretation and writing the manuscript. MMM assisted with the study design and writing of the manuscript. DDC participated in study design and provided background data on HC-030031. All authors have read and approved the final manuscript.
